# Abuse of Anabolic-Androgenic Steroids as a Social Phenomenon and Medical Problem—Its Potential Negative Impact on Reproductive Health Based on 50 Years of Case Report Analysis

**DOI:** 10.3390/jcm13195892

**Published:** 2024-10-02

**Authors:** Monika Skrzypiec-Spring, Julia Rozmus, Gina Abu Faraj, Kinga Brawańska-Maśluch, Krzysztof Kujawa, Adam Szeląg

**Affiliations:** 1 Department of Pharmacology, Wroclaw Medical University, 50-345 Wroclaw, Poland; 2 Statistical Analysis Centre, Wroclaw Medical University, 50-367 Wroclaw, Poland

**Keywords:** reproductive health, reproductive endocrinology, infertility, reproductive medicine, lifestyle, anabolic-androgenic steroids

## Abstract

**Background/Objectives:** Illegal anabolic-androgenic steroids are a significant lifestyle factor in infertility. The aim of our study was to analyze clinical cases resulting from their use for their frequency, geographical location, dynamics, substances used, the age and gender of the users, and the types of clinical complications. **Methods:** Publications were obtained by searching PubMed for the following terms: ‘anabolic-androgenic steroids’ and ‘clinical case’. Publications from 1973 to 2022 were qualified for the analysis. **Results:** An increasing trend in the number of clinical cases resulting from the use of steroids, as well as the number of substances used simultaneously, was observed. The substances changed over the decades, but in the last 20 years, testosterone, nandrolone, stanozolol, methandienone, trenbolone, and methenolone have predominated. Cardiological side effects predominated in each period, with a continuous increase in their occurrence. The most common among these were myocardial infarctions and hypertrophic cardiomyopathy. The next most numerous adverse events involved psychiatric, endocrinological, hepatic, and oncological problems. We demonstrated a possible relationship between the use of individual steroids and medical issues; the strongest associations were between testosterone and endocrine complications, and methylstenbolone and hepatic complications. **Conclusions:** There has been an increasing trend in case reports describing serious health problems associated with the use of anabolic-androgenic steroids, a tendency to use several substances simultaneously, and a preferential use of substances with a high potential of causing serious side effects. These phenomena mainly concern men, with an average age of 30, and the health problems that dominate in clinical case reports—including serious cardiological, psychiatric, endocrinological, hepatic, and oncological diseases—may potentially affect reproductive health and pose a challenge for reproductive medicine.

## 1. Introduction

The incidence of infertility in couples of reproductive age is approximately 15% [1]. The contribution of male factors to infertility, depending on the source, ranges from 20% to 70%, and the percentage of infertile men ranges from 2.5% to 12%. [1]. Globally, it is estimated that between 48 million couples and 186 million people of reproductive age suffer from infertility [2]. Currently, the world’s average annual human population growth rate is 1.2% [3]. This increase is due to improved living conditions, health, and life expectancy [4]. On the other hand, a reduction in fertility has been observed. A global study conducted in 2015 showed that about 30 million men were infertile, with higher infertility rates in areas of Africa and Eastern Europe [5]. The phenomenon of generational replacement may not occur when there are approximately 2.1 children per woman, which is necessary for generational replacement. One major factor reducing fertility is the presence of endocrine disruptors, which are dominated by environmental pollution, including heavy metal contamination and other natural or man-made chemicals [6].

The hormonal system regulates spermatogenesis and plays a key role in male fertility.

Testosterone (T), the main androgen and the main hormone secreted by the testicles, is necessary for both the initiation and maintenance of spermatogenesis. The progression of spermatogenesis beyond the stage of meiosis and the production of mature sperm strictly depends on T and does not occur in its absence, leading to infertility [7]. In men, T is produced by Leydig cells in response to stimulation by the luteinizing hormone (LH). It, in turn, is secreted from the pituitary gland in response to the pulsatile release of the gonadotropin-releasing hormone (GnRH) from the hypothalamus. The follicle-stimulating hormone (FSH), the second gonadotropin secreted by the pituitary gland, is also necessary to maintain fertility. Although FSH plays an essential role in preparing for spermatogenesis before puberty, it is also involved in the regulation of spermatogenesis after puberty [8]. During the prepubertal, fetal, and early postnatal periods, FSH regulates Sertoli cell proliferation. Subsequently, it promotes the differentiation of Sertoli cells and is responsible for spermatogonia pool maintenance, differentiation, and survival. In adult males FSH is necessary for the onset of meiosis, facilitating entry into meiosis, and positively regulating spermatocyte survival.

Factors that disrupt the hypothalamic–pituitary–testicular axis have a negative impact on reproductive endocrinology, leading to infertility [8]. One factor is exogenous T. T was independently synthesized by Adolf Butenandt and Leopold Ruzicka, which earned them the Nobel Prize in Chemistry in 1939 [9]. Initially, T was used in the form of subcutaneous pellets and orally as 17α-methyltestosterone. In the 1950s, testosterone enanthate was introduced in the form of an intramuscular injection. The 1950s and 1960s were a period of research on androgens aimed at enhancing their anabolic effects on muscles or bones to treat cachexia and osteoporosis, or to enhance erythropoietic for the treatment of anemia. In 1956, 256 androgenic steroids were described, and by 1976 the number exceeded 1000 [10]. Since then, T and anabolic androgenic steroid(s) (AAS) have become widely used to improve athletic performance. It has not been possible to produce anabolic compounds without the ability to induce androgenization; for this reason, they are now rarely used in clinical medicine, and they continue to be illegal doping agents in sports [9].

The influence of exogenous T and AAS on fertility has been the subject of many studies. A comprehensive review of 32 publications covering 671 AAS users by Mulawkar et al. showed that exogenous T and/or AAS have a negative effect on gonadotropin levels, which persists for 3–6 months after AAS discontinuation, resulting in lower sperm motility and testicular size [11].

The use of exogenous T and/or AAS has a negative impact on male fertility by inhibiting the hypothalamic–pituitary–gonadal axis and reducing spermatogenesis. It can also cause structural changes to the testes, such as morphological changes in Leydig cells [12], a decrease in their number, anomalies in the meiotic process, and genetic damage [13,14]. Impaired fertility and abnormal semen parameters may persist for a long time after discontinuing exogenous T and/or AAS [14]. In light of this, the illegal use of T and AAS is a significant lifestyle factor in male infertility. The use of AAS by women is less common, with a lifetime prevalence of 1.6% [15]. Therefore, the side effects it causes, including the effect on fertility, are less researched, and data on this subject are mainly derived from surveys or scientific studies on small research groups, making it difficult to draw conclusions.

Survey research shows that side effects of using AAS (as perceived by women) include menstrual irregularities, clitoral enlargement, deepening of the voice, an increase in facial hair, and aggressiveness [16,17,18].

Regarding menstrual alterations, the main side effects reported during AAS use are delayed menarche, oligomenorrhea, secondary amenorrhea, dysmenorrhea, and anovulation [19].

Data regarding the endocrine effects of AAS use in women who self-administer T and anabolic steroids are very limited. In the menstrual cycle, serum levels of T and androstenedione increase gradually during the follicular phase, reaching their peak in the pre-ovulatory phase [20,21]. Then, during the late luteal phase, the androstenedione level further increases but serum T concentrations remain at a constant level [20,21]. In women using AAS, there is an increase in serum T levels, a significant compensatory decrease in the sex hormone-binding globulin, and a decrease in the thyroid-binding protein [22]. An excessive increase in male hormones and a decrease in hormone-binding globulins can disrupt the hormonal balance and lead to anovulation and irregular periods, but there is no scientific data to explain the exact mechanisms responsible. Also, to our knowledge, there are no scientific studies on the impact of AAS on the structure of female gonads.

In addition to the direct impact on fertility, in both sexes, androgen abuse leads to many other side effects that may indirectly affect fertility.

Hepatotoxicity is the most serious side effect associated with the use of 17α-alkylated androgens and SARMs [23,24,25], which may lead to various manifestations, including liver tumors, adenoma, carcinoma, cholangiocarcinoma, angiosarcoma, hepatic peliosis, and cholestasis [26,27]. Peliosis hepatis causes enlargement of the liver and spleen and may lead to fatal bleeding [28]. Other serious side effects include cerebral or deep vein thrombosis, pulmonary embolism, and cerebral hemorrhaging [29]. Significant side effects include cardiovascular diseases, such as hypertension, cardiomyopathy, and dyslipidemia [30]. Sharing needles may result in HIV infection and hepatitis [31,32]. Tendon ruptures and rhabdomyolysis are also complications associated with androgen abuse [33]. Psychological effects are also among the most significant side effects of androgen abuse. They affect one’s mood in a dose-dependent manner and cause hypomania [34]. Androgen abuse is associated with increased impulsivity, aggression, violence, or dysphoria, including depression and even psychosis. Behaviors such as bigorexia, tolerance, and withdrawal symptoms are typical for people abusing androgens [34].

According to Refs. [23,24,25,26,27,28,29,30,31,32,33,34], the scale and characteristics of illegal AAS use, as well as the dynamics of change taking place in sports doping, are very difficult to reliably estimate due to the illegality of purchasing and using AAS. Attempts to assess this phenomenon are mainly based on anonymous surveys. The analysis of related clinical cases allows for a different look at this problem and obtaining other data. Therefore, our study analyzes clinical case reports resulting from the illegal use of AAS from the period between 1973 and 2022. It specifically looks at the frequency of clinical cases resulting from the use of AAS, their location, the dynamics of changes in these parameters, the type of ASS used, the age and gender of people using them, and the types of clinical complications.

## 2. Materials and Methods

Publications were obtained by searching PubMed. Search terms included ‘anabolic-androgenic steroids’ and ‘clinical case’. Publications from the period 1973–2022 were qualified for the analysis. The last search date was 10 June 2024. Only publications for which it was possible to obtain access to the full text of the article were included in the analysis. As this paper is based on an exploratory analysis, the equal weighting of cases was not assessed. Full-text publications were analyzed in terms of the year of publication, place of occurrence of the clinical case, type of AAS, age, gender, and medical diagnosis. The analyzed 50-year period was divided into 10-year sub-periods. The division was used to balance the need for larger sample sizes within each analyzed period and a sufficient number of time units to determine linear trends and changes in the direction of trends. We considered a 10-year period to be appropriate.

The analysis of trends in changes in the number of publications, types of substance used, and patient age was performed using local polynomial regression fitting (LOESS) with a 95% confidence interval. The effects of the continent on the number of AAS types used were analyzed using the Kruskal–Wallis test. The relationships between the patient’s age, continent, and decade were analyzed using bootstrapped ANOVA (due to the data distribution deviating from normal). The relationships between categorical variables were analyzed using the exact Fisher’s test (due to many small observations, i.e., less than five). Since multiple tests were performed on the same dataset, the analysis was considered as testing a family of hypotheses, and *p*-values were adjusted using the Benjamini–Hochberg correction to control the false discovery rate. The Kruskal–Wallis test and descriptive analyses were performed using Statistica 13 (TIBCO Software Inc., Palo Alto, CA, USA, 2017) and the other analyses (Fisher test, Benjamini–Hochberg correction, LOESS, bootstrapped ANOVA) were performed using the R-environment version 4.3.2 (R Core Team. _R: A Language and Environment for Statistical Computing. R Foundation for Statistical Computing, Vienna, Austria, 2023)

## 3. Results

### 3.1. The Number of Papers Per Year from 1973 to 2022

#### A Total of 397 Cases Were Analyzed

**Figure 1:**
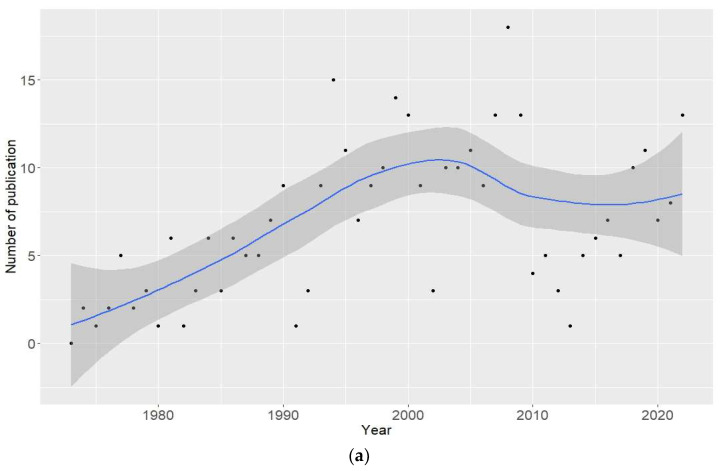

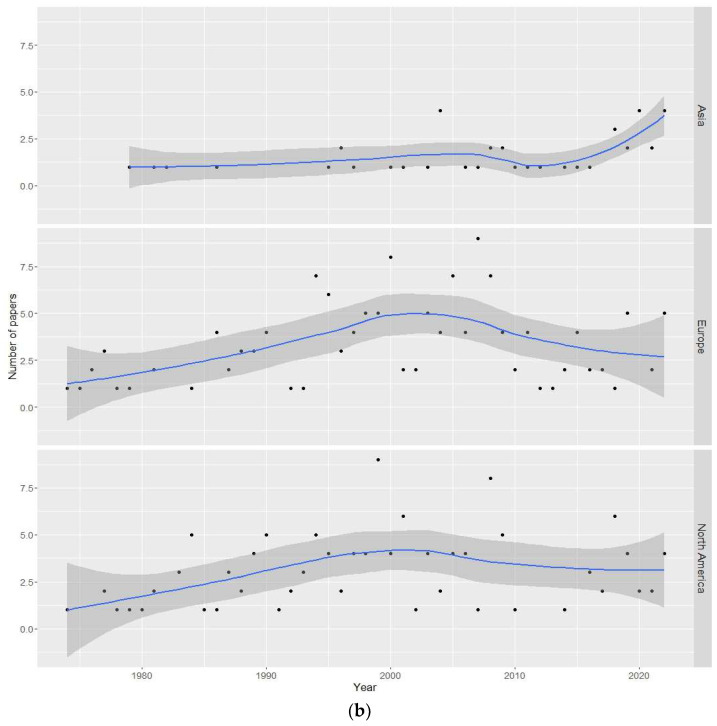
The number of clinical case reports resulting from the use of anabolic-androgenic steroids. (**a**) Number of papers per year during the period 1973–2022. (**b**) Number of publications per continent per year during the period 1973–2022. Gray area—95% confidence interval of the curve.

From 1973 to 2022, a weak upward trend in the number of clinical case reports resulting from the use of anabolic-androgenic steroids was observed, especially during the period 1973–2002 (Figure 1a). When divided into continents, this trend was particularly visible in Asia in 2013–2022, with a stable number of publications in Europe and the United States. In Europe and the United States, an upward trend was observed in the years 1973–2002, with a stable number of publications in Asia pointing to the stabilization of AAS use in Europe and the United States in the last decade, while the scale of this phenomenon has spread to Asia. (Figure 1b).

### 3.2. The Number of Anabolic-Androgenic Steroid Types Per Clinical Case

**Figure 2:**
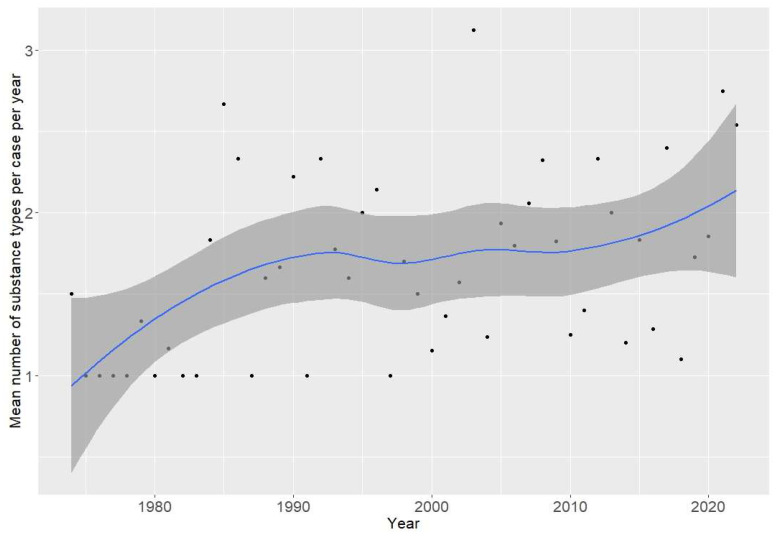
The number of anabolic-androgenic steroid types per clinical case during the period 1973–2022. Gray area—95% confidence interval of the curve.

From 1973 to 2022, there was a weak upward trend in the number of substance types per clinical case (Figure 2).

### 3.3. The Number of Anabolic-Androgenic Steroid Types Per Continent

**Figure 3:**
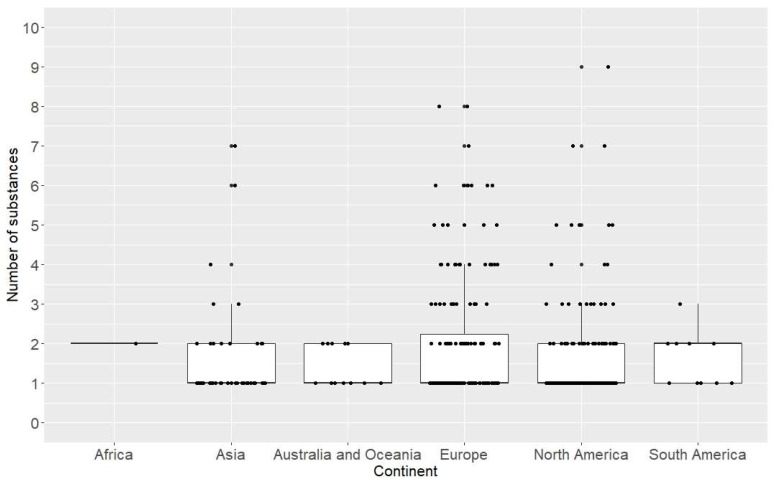
Number of anabolic-androgenic steroid types per case per continent. Comparison of the number of anabolic-androgenic steroid types per continent between 1973 and 2022. Boxes and whiskers represents medians, 1st and 3rd quartiles, and the ranges without outliers. The dots are the number of AAS per case.

**Figure 4:**
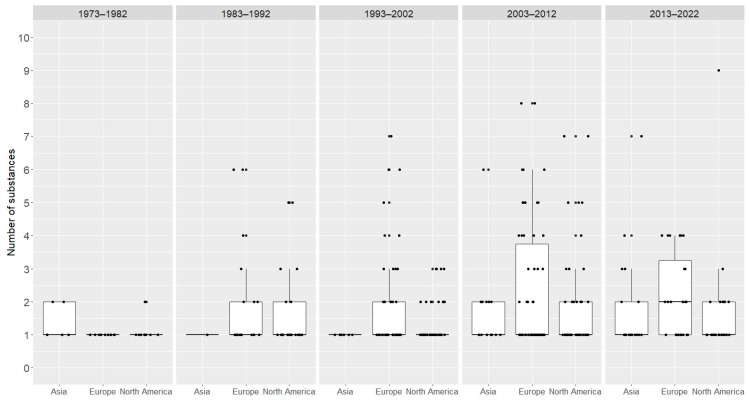
Comparison of the number of anabolic-androgenic steroid types used in Asia, Europe, and the United States in the decades since 1973. Boxes and whiskers represents medians, 1st and 3rd quartiles, and the ranges without outliers. The dots are the number of AAS per case.

There were no significant differences in the number of anabolic-androgenic steroid types between continents (Kruskal–Wallis test: H (5, 395) = 7.09, *p* = 0.21) (Figure 3). However, the dynamics of change in these amounts were observed in Europe, the United States, and Asia, with a constant increase in this amount since 1992 in Asia. In Europe and the United States, an increase was observed between 1992 and 2002, after which, there was a decline in the number of anabolic-androgenic steroid types used (Figure 4).

### 3.4. The Ages of People Involved in Clinical Cases

**Figure 5:**
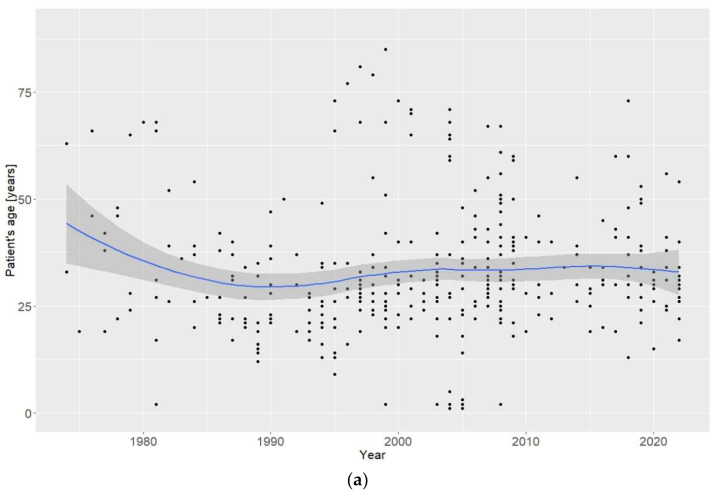

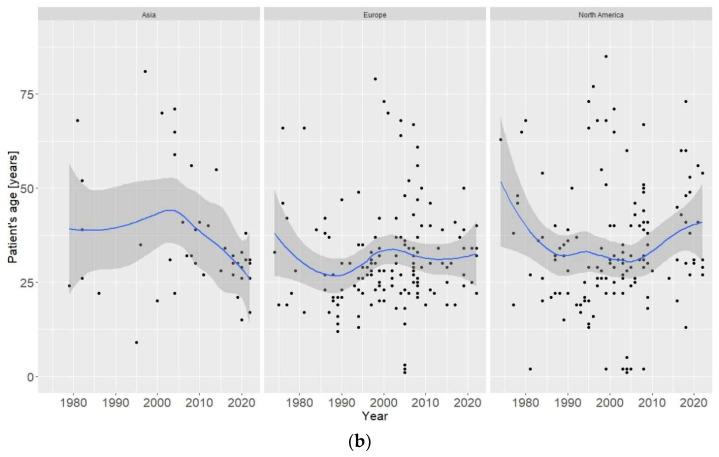
The age of people using anabolic-androgenic steroids involved in clinical cases. (**a**) The average age of people involved in clinical cases over the period 1973–2022. (**b**) Comparison of the average age of people involved in clinical cases between continents. Gray area—95% confidence interval of the curve.

**Figure 6:**
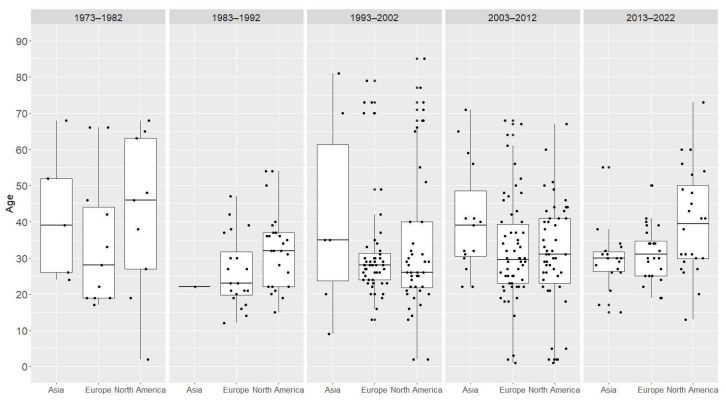
The age of people using anabolic-androgenic steroids involved in clinical cases per continent. Boxes and whiskers represents medians, 1st and 3rd quartiles, and the ranges without outliers. The dots represent the individual patient’s age.

The median age of participants involved in clinical cases was 30 (Q1 = 23.5, Q3 = 39.0). Although no discernible trend in the average age of participants involved in clinical cases (from the entire group) was observed over the period 1973–2022, a notable difference emerged between Asia (decrease) and North America (increase) within the last two decades (Figure 5a,b). The decreasing age of AAS users, along with the increase in AAS use in Asia over the last decade, indicates that AAS use represents an escalating social and public health problem on that continent. There was no statistically significant effect of time (decades), continent, or the interaction between time and continent (bootstrapped ANOVA: *p* = 0.168, *p* = 0168, *p* = 0.087) on the age of patients (Figure 6).

### 3.5. The Gender of People Using Anabolic-Androgenic Steroids Included in Clinical Case Reports

**Figure 7:**
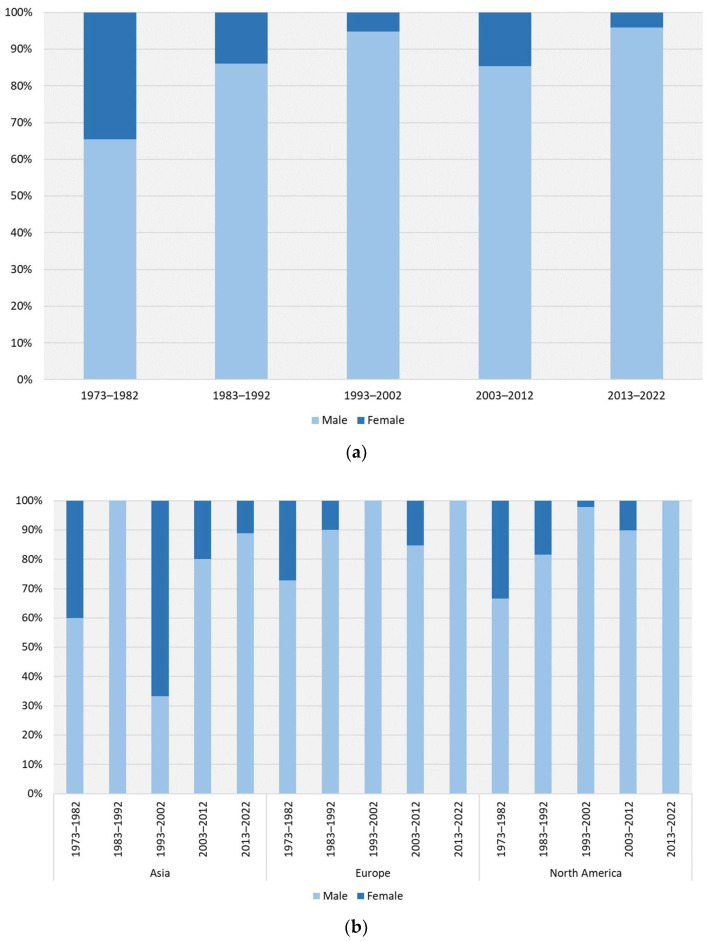
The gender of people using anabolic-androgenic steroids involved in clinical cases per continent. (**a**) The changes in all continents together. (**b**) The changes in continents with the largest number of cases.

Men predominate among all people using anabolic-androgenic steroids in the case reports, even when divided into continents and decades. Their percentage amounted to 88.2% of the whole group. The percentage was 75% in Asia, Australia, and Oceania; it was 91.7% in Europe. Percentages ranged from 65.4% to 95.9% across the decades. The effects of both the continent and decade on the sex proportion were statistically significant (Chi-square test: χ^2^ = 10.42, *p* = 0.0055, and χ^2^ = 24.14, *p* < 0.0001, respectively). Additionally, a reduction in the share of women was observed from 34% in the period 1973–1982 to 4% over the period 2013–2022 (Figure 7a). In terms of continent, the share of women using AAS in Asia is much higher than in Europe and North America, which is also visible when broken down by decade, but the sample sizes in Asia are quite small, which is another disturbing phenomenon that may indicate that AAS use is a significant social and public health problem in that region (Figure 7b).

### 3.6. The Use of Particular Substance Types, Regardless of Whether They Were Used Individually or Many Substances at the Same Time Were Used

Table 1 presents the names of anabolic-androgenic steroids used in the analyzed clinical cases and the number of cases in which individual substances were used during the period 1973–2023, divided into decades. Table 2 shows the percentage of anabolic-androgenic steroids used in the analyzed clinical cases in individual decades. Clinical cases associated with the use of T predominated in all time intervals.

There was a gradual reduction in the percentage of methandienone used, a sharp decrease in the percentage of oxymetholone used between 1973 and 1982 and 1983–1992, with a subsequent slight increase followed by a gradual decrease, and a sharp decrease in the percentage of fluoxymesterone used from 1983. An increase in the percentage of nandrolone use has been observed since 1983.

In the last two decades, the most common AAS mentioned in clinical case reports, apart from testosterone, include nandrolone, stanozolol, methandienone, trenbolone, and methenolone.

After dividing by gender, it was shown that, with women, the use of T predominated (*n* = 17, 35.4%). Oxymetholone and danazol were in second place. Stanozolol was ranked third, followed by methandienone and methenolone. Regarding clinical cases involving the use of testosterone by women, the largest number occurred within the 2003–2012 time period. With men, the distribution of individual substances described in the clinical cases aligns with the overall distribution without dividing by gender (χ^2^ = 3.93, *p* > 0.9999).

### 3.7. The Use of Particular AAS Types Individually

Table 3 presents a numerical summary of clinical cases of substances used individually in each decade within the 1973–2022 period. Table 4 shows the percentages of clinical cases of substances used individually in each decade within the 1973–2022 period.

In the last decade, the most common AAS types used in clinical case reports were testosterone, nandrolone, and methylstenbolone.

### 3.8. The Use of Particular Types of ASS in Combination with Several Substances

Table 5 presents a numerical summary of clinical cases with substances used in combination with several AAS types at the same time in each decade of the period 1973–2022. Table 6 shows the percentage of clinical cases with these substances.

In the last decade, the most common AAS types used in combination with several substances were testosterone, nandrolone, stanozolol, trenbolone, methandienone, and methenolone.

### 3.9. Types of Diseases Resulting from the Use of AAS Covered by Clinical Case Reports

Among the various types of diseases resulting from the use of AAS during the analyzed 50-year period, the most common were cardiological, psychiatric, endocrinological, hepatic, oncological, dermatological, andrological, neurological, hemorrhagic, thromboembolic and orthopedic complications. Table 7 presents the distribution of medical problems among all cases.

Clinical cases involving cardiac complications were the only ones that showed continuous growth. From 1993 to this day, they have been dominant following the use of AAS.

The number of clinical cases involving psychiatric and endocrine complications showed a consistent increase until 2012 and then decreased in the last decade examined. The number of hepatic complications remained at a similar level, except for the period 1983–1992 when the number was lower. The number of oncological complications was the highest during the period 1993–2002, after which it decreased and has remained at a constant level since then. The number of dermatological complications increased after 1982 and remained stable until 2012, but then decreased. The number of andrological complications showed a consistent increase until 2012; during the period 2013–2022, no clinical cases of this type were described. The number of neurological complications showed a consistent increase until 2012, after which there was a slight decrease. The number of hemorrhagic and thromboembolic complications remains at a similar level. The number of orthopedic complications increased, reaching a peak between 1993 and 2012, with a subsequent reduction.

In the last decade under study, the dominant complications were cardiological, hepatic, and oncological, followed by neurological, psychiatric, thromboembolic, endocrinological, and gastroenterological. The described changes are presented in Table 8.

When it comes to the percentage share of individual types of side effects in relation to the total number of cases described in a given period, cardiac complications show an increasing tendency, and hepatic, oncological, hemorrhagic, and thromboembolic complications show a decreasing tendency (Table 9).

Cardiological, endocrine, infectious, hepatic, andrological, and oncological complications were involved in various disease entities, in which myocardial infarction, virilization, cholestasis, hypertrophic cardiomyopathy, liver cancer, and male infertility were the most frequent (Table 10).

After dividing by gender, it was observed that, in women, clinical cases related to endocrine disorders, mainly virilization, predominated. Other prevalent conditions included endocrinological, oncological, cardiological, psychiatric, and dermatological diseases. With men, the distribution of diseases described in clinical cases was similar to the overall distribution (without dividing by gender), except for the absence of gynecological problems (obviously) in males and fewer endocrinological problems (6.8% vs. 9.6% in females) (χ^2^ = 7.35, *p* > 0.9999).

### 3.10. Dependence of a Given Type of Side Effect on the Type of AAS—When Used Individually

The test examining the dependence of a given type of disease on the substance used identified the AAS most frequently reported to cause a given complication. Due to the increased risk of error in such testing, the Benjamini–Hochberg correction was used. The statistically significant results are presented in Table 11. All results are presented in Table S1.

The table presents the relationships as significant, at least without using correction for controlling the I-type error. The occurrence (%) is calculated in relation to the number of patients with the given type of side effect. P adj. means the *p*-value calculated using the Benjamini–Hochberg correction for controlling the I-type error.

The results indicate the possibility of a relationship between the use of androstenedione and andrological complications; nandrolone and cardiological complications; testosterone and dermatological and endocrine complications; danazol and hematological complications; methandienone and hemorrhagic complications; methylstenbolone, oxymetholone and testosterone and hepatic complications; methylstenbolone and stanozolol and nephrological complications; dehydroepiandrosterone and neurological complications; methyltestosterone and oncological complications; stanozolol and orthopedic complications; dehydroepiandrosterone and methandienone and psychiatric complications; and fluoxymesterone and testosterone and thromboembolic complications, with the probability of this dependence being the strongest in cases of testosterone and endocrine complications and methylstenbolone and hepatic complications. In other cases, there is a probability that the indicated dependencies are false.

Additionally, a relationship was demonstrated between the use of many substances simultaneously and cardiac, endocrine, and metabolic complications (Table 12).

## 4. Discussion

### 4.1. The Use of AAS as a Social Phenomenon over 50 Years

Data obtained from the analysis of clinical case reports of AAS use over 50 years (since 1973) indicate an increasing trend in the number of publications.

A similar trend appears in the frequency of AAS use as declared in anonymous surveys. Skrzypiec-Spring et al. in 2024. and Montuori et al. in 2021, in a cross-sectional analysis of data from anonymous surveys in Wrocław (Poland) and Naples [35,36], showed that the frequency of use of T and AAS in gyms was approximately 35% (35.2% and 35.5%, respectively).

Data from 1993 by Korkia and Stimson, who studied the use of AAS among strength trainees in 21 gyms in the UK, showed that 6% of men surveyed used them during the study, and 9.1% had used them at some point [37]. A meta-analysis of 271 articles, conducted by Sagoe et al. in 2014, showed that the global prevalence of the use of these substances in recreational sports was 18.4% [15]. The combination of data and the results of our study clearly indicates an increasing trend in the illegal use of AAS over the last 30 years.

Men dominated among the AAS users in the clinical case reports. The share of women in 2013–2022 was 4%. This percentage is more than twice as high as that reported in surveys [15] and may indicate that women are more susceptible to significant side effects.

Another trend that our data analysis found involved the number of substances used at one time. Namely, there was a weak trend toward an increase in the number of AAS types per clinical case. The phenomenon of increasing the number of AAS types is very unfavorable because the number of substances used may increase the number of undesirable effects. To our knowledge, there are no publications that characterize the dynamics of changes in the AAS amount at the same time, and our publication is the first to address this aspect of the illegal use of anabolic-androgenic steroids (although, according to publications from recent years, most people abusing AAS have adopted a polypharmacy strategy to increase their effects) [38,39,40]. According to current data by Smit et al., a median of five different AAS types is used during one period of AAS usage, with a range of one to eleven different substances [40]. According to our data, a single AAS was used in 257 (64.7%) cases, and several substances were used simultaneously in 140 (35.3%) cases.

The trend may be attributable to several factors. The use of AAS can lead to muscle dysmorphia and dependencies, which predispose individuals to increase both the doses and the number of preparations to enhance their effects. Muscle dysmorphia, in turn, is related to social pressures to become both more muscular and leaner, likely influenced by media, which has undergone rapid development in the last fifty years [41,42].

Both trends are very unfavorable, especially since they concern men of reproductive age (we showed that the average age of individuals participating in the clinical case reports was 30). Our results are consistent with those obtained from surveys on the use of AAS. Skrzypiec-Spring et al. showed that the typical man using T and AAS is between the ages of 26 and 35; men aged 26–30 use T and AAS 2.62 times more often than men aged 18–25, and men aged 31–35 years use it 3.24 times more often [35]. Our results are also consistent with those of other studies. Al Hashimi et al. showed that AAS was most frequently used by people aged 20–30 years (74.83%) and 30–40 years (13.25%); the findings were based on an online survey conducted among urologists, andrologists, and endocrinologists [43]. Similarly, Montuori et al. (2021) showed that the average age of people using AAS was 32.42 years old [36].

### 4.2. The Pattern of AAS Use over 50 Years

Over the analyzed 50-year period, a decreasing trend was observed in the number of clinical case reports related to the use of oral 17-alpha-alkylated steroids (methandienone, oxymetholone, and fluoxymesterone). This trend may be related to the fact that they possess a high potential for hepatotoxicity, which limits their use. Interestingly, this trend was not observed in relation to another steroid from this group, stanozolol, which is also characterized by high hepatotoxicity. However, it is considered an AAS with a low potential of causing gynecomastia. The second trend observed was the increase in the number of clinical cases resulting from the use of nandrolone. Although animal studies indicate that it may be aromatized to estradiol, there are no studies on humans, and it is considered a substance with the lowest potential of causing gynecomastia. Analyzing the above trends, it can be assumed that the potential to cause gynecomastia and hepatotoxicity significantly influences the choice of AAS by users. This may be related to the ease of recognizing these undesirable symptoms, the fear of unfavorable cosmetic effects, and the need to reduce the doses and effectiveness of illegal support due to side effects.

Among women, clinical case reports were mainly associated with the use of T. According to other sources, the most frequently used AAS is oral oxandrolone because it is considered to be less androgenic than T [44]. As virilization is the most common disease described in women using AAS, it can be assumed that the results we obtained regarding the AAS types described in clinical cases do not reflect which substances women use most often, but which substances most often cause them to suffer from side effects.

Among the AAS types reported in clinical cases, T predominated in all decades. In the last two decades, the most common AAS types in clinical case reports, apart from T, include nandrolone, stanozolol, methandienone, trenbolone, and methenolone. Our results partially coincide with those obtained by Bates et al. [45]. They showed that the AAS types used by respondents were dominated by various T esters, trenbolone, nandrolone, drostanolone, boldenone, and stanozolol. In turn, according to Smit et al., the most commonly used AAS types included testosterone (96% of the users), trenbolone (52%), drostanolone (39%), and boldenone (38%) [39].

The difference in the lack of methandienone and methenolone in the results of Bates et al. and Smit et al. may result from the fact that their studies were conducted in Great Britain and the United States, respectively, while the clinical case reports we analyzed involved all continents and may reflect different patterns in AAS usage between countries [39,45]. However, percentage differences are most likely due to the fact that a high frequency of use of a substance does not always correspond to a high frequency of side effects that can be described as a clinical case. This especially applies to drostanolone and boldenone, which are among the most frequently used AAS types (according to the studies cited above) and are not the most frequently described AAS types in clinical cases. In turn, the opposite situation may apply to methandienone, which is known for its high hepatotoxicity and is often described in clinical cases, but is not listed among the most frequently used AAS types according to Bates et al. and Smit et al. [39,45].

### 4.3. The Types of Complications Associated with the Use of AAS and the Dynamics of Their Changes over 50 Years

The tendency to use many substances simultaneously, along with changes in the type of ASS used, certainly impacts the types of complications. These complications, in turn, may indicate the involvement of AAS use in reproductive issues. To our knowledge, our study is the first attempt to assess the scale of complications associated with the use of AAS types and the dynamics of change over a 50-year period (starting in 1973).

Since 1983, the leading complications associated with the use of AAS have been cardiac complications, which are also the only ones with a consistently increasing rate of change. The most common of these include hypertrophic cardiomyopathy and myocardial infarction. Cardiovascular risk is especially associated with long-term AAS use [46,47]. Although these cardiac complications do not directly impact fertility, they may have a significant indirect effect on reproduction by impacting standard treatments (e.g., beta-blockers), which can impair sexual function (and, thus, reduce sexual activity). Moreover, these complications might impact couples’ decisions about procreation, particularly when it comes to severe cardiac diseases (faced by men) with an uncertain prognosis. The increasing number of cardiac complications in young men using AAS is also very disturbing for other social reasons because these diseases limit the ability of young men to work, limit their social life, and constitute a growing healthcare burden.

Psychiatric complications related to psychosis and aggressive behavior are very disturbing problems that may affect reproduction and are some of the most common complications of AAS use. Impaired memory and changes to cognition are related to long-term use of AAS [48]. Also, it has been shown that long-term use of high-dosage AAS is associated with structural changes in the brain [49,50,51,52]. Although the number of these clinical cases has decreased in the last decade, there has been an upward trend in the previous 40 years (remaining in fifth place in terms of the number of cases described). These complications may indirectly affect reproduction through the drugs used and negatively impact relationships and decisions about procreation. There is even the possibility of people turning to crime. Endocrine complications, which include gynecomastia and testicular atrophy in men and virilization in women, and show similar dynamics to psychiatric complications, may, however, have a direct negative impact on reproduction. Hepatic complications, which are currently the third most common in clinical case reports, may affect fertility directly by affecting the metabolism of sex hormones and sex hormone-binding proteins, as well as indirectly, due to chronic severe diseases. Oncological complications, mainly liver cancer, are also very significant from reproductive and social perspectives. Although the number of clinical cases reported regarding these complications has decreased since 2012, they currently constitute the third most common medical problem associated with the use of AAS. These complications may indirectly affect reproduction due to the direct impact of cancer treatment on male gonadal function, as well as by influencing couples’ decisions about procreation when faced with cancer and its uncertain prognosis in men.

The number of clinical cases involving permanent infertility or permanent impotence has shown an upward trend for four consecutive decades, which is surprising considering the direct negative impact of AAS on spermatogenesis and sexual function. Surprisingly, no clinical cases regarding these conditions have been reported in the last decade. Perhaps this phenomenon is related to the fact that the direct effect of AAS on fertility is a well-known problem, and clinical cases should concern atypical medical problems. Neurological complications are also significant when it comes to the indirect effects on reproduction and primarily include ischemic and hemorrhagic strokes, as well as thromboembolic events and hemorrhagic complications, mainly resulting from the rupture of benign tumors.

### 4.4. The Relationship between the Individual Complications of AAS Use and the Type of Substance Used

Our analysis of clinical case reports allowed us to assess the dynamics and frequency of clinical complications of AAS types as well as the relationship between individual complications of AAS use on the type of substance used. The number of publications on this problem is limited to single publications regarding only certain types of complications, and our publication is the first attempt to assess the relationship between all types of complications described over 50 years, with all AAS types used in the described cases.

The results indicate a high probability of a relationship between the use of T and endocrine complications in the form of gynecomastia and testicular atrophy in men or virilization in women, as well as methylstenbolone and hepatic complications.

The relationship between T and androgenic effects, strong inhibition of pituitary function, and aromatization to estradiol is apparent. These are well-known and documented side effects of T (the goal in the synthesis of new AAS was to avoid these side effects).

Methylstenbolone belongs to 17α-alkylated AAS. The 17α-alkyl substitution delays the metabolism of (and contributes to the high potential for) hepatotoxicity. In addition to methylstenbolone, a substance–hepatotoxicity relationship has been demonstrated for another 17α-alkylated AAS, oxymetholone, and T, but with these substances, there is the possibility that the relationship is spurious. We have not found any publications that could explain why this relationship was demonstrated for these particular two representatives of 17α-alkylated AAS, and the hepatotoxicity of 17α-alkylated AAS is poorly understood and attributed to oxidative stress [53]. In animal models, a correlation between the susceptibility of the mouse liver to injury and inflammatory response (mediated by androgen receptors) has been demonstrated [54]. Both methylstenbolone and oxymetholone belong to dihydrotestosterone derivatives, with a very high affinity for the androgen receptor, which may contribute to their high potential to cause hepatotoxicity. In turn, T in the liver is metabolized to dihydrotestosterone by type 1 5α-reductase, which is most prevalent in extra-prostatic tissues, like the liver [55].

The results indicate the possibility of a relationship between the use of androstenedione and andrological complications, such as infertility and impotence. The scientific data on this topic are also limited. For the most part, androstenedione use resulted in sperm count reduction, impotence, gynecomastia, and prostate enlargement, although it is mainly associated with carcinogenicity [56].

We also demonstrated the possibility of a relationship between the use of nandrolone and cardiological complications. Although there are many publications on the mechanisms of AAS cardiotoxicity, we did not find any publications that assessed the cardiotoxic potential of individual substances. A systematic review of the literature on nandrolone showed that cardiac complications were the second most frequently reported side effect associated with its use [57]. In turn, nandrolone is characterized by dose-dependent cardiotoxicity, and no cardiotoxicity is observed at pharmacological doses [57,58]. Despite the possible relationship between nandrolone and cardiac complications, there is no clear evidence or scientific evidence that could confirm this.

The relationship between the use of danazol and hematological complications in the form of increased hematocrit is apparent, as the effect of danazol on the hematopoietic system is used in the treatment of hematological diseases [59,60].

The relationship between methandienone and hemorrhagic complications, mainly resulting from the rupture of liver adenomas, seems to be related to its chemical structure, as it belongs to 17α-alkylated AAS. The mechanism by which the androgens activate hepatocyte growth involves the engagement of intracellular androgenic steroid receptors. [61]. According to other theories, AAS induces the growth of hepatic adenomas after the aromatization of androgen to estrogen [39]. In addition to being a 17α-alkylated AAS, methandienone is subject to aromatization and exerts moderate estrogenic activity, which may provide theoretical grounds for its great potential when it comes to benign liver tumors, and complications such as their rupture. The same mechanism may explain the relationship between methyltestosterone and oncological complications, among which, liver cancer predominated.

Another relationship revealed in our study is between the use of methylstenbolone, stanozolol, and nephrological complications. AAS may cause glomerular and interstitial damage by direct renal toxicity. Moreover, 17-α-alkylated AAS may also produce cholestasis and result in bile acid nephropathy [61]. Also, apart from hepatic tumors, there are isolated reports of Wilms tumors. In addition, they may cause hypercalcemia and nephrocalcinosis [62]. Although there is no data on the nephrotoxic potential of individual AAS, these mechanisms may explain the relationship between these two representatives of 17-α-alkylated AAS and nephrotoxicity.

Similarly, to date, there is no scientific data on the relationship between psychiatric complications and the use of specific AAS types. The mechanisms underlying aggressive behaviors that predominated among psychiatric complications are poorly understood. Animal studies have shown that male mice lacking functional androgen receptors did not display enhanced aggression following the administration of AAS, which supports the involvement of the androgen receptor in generating aggressive behavior [63]. However, the estrogen receptor has also been shown to mediate aggression in males. Exogenous estrogen administration in male mice lacking the androgen receptor and testosterone also increased aggression [64]. Furthermore, activation of estrogen receptor α is associated with increased aggression, while activation of estrogen receptor β is associated with decreased aggression [65]. In our work, we demonstrated the possibility of a relationship between psychiatric complications and methandienone and dehydroepiandrosterone. Methandienone shows moderate estrogenic activity, and dehydroepiandrosterone in animal studies increased the androgen receptor and aromatase mRNA expression in the brain, which, in both of these substances, may explain their potential to cause aggressive behavior (and the demonstrated relationship) [66].

We also demonstrated a possible relationship between dehydroepiandrosterone and neurological complications, including hemorrhagic and ischemic strokes. There are no scientific data confirming such a relationship. Data regarding the effect of this AAS indicate that dehydroepiandrosterone administration reduces plasma PAI-1 and tPA antigen concentrations in men and enhances endogenous fibrinolytic potential, which could be related to the relationship we demonstrated [67]. For this reason, a relationship between dehydroepiandrosterone and thromboembolic complications is unlikely. There is no scientific data on such side effects of this AAS. Testosterone is known for its thromboembolic properties. It produces erythrocytosis due to a multifactorial process, including the stimulation of erythropoietin, and the suppression of hepcidin, ferritin, and estradiol, which increases hematopoietic telomerase [68]. However, there are no scientific data that could confirm the relationship between fluoxymesterone and thromboembolic complications.

Although tendon ruptures are a common complication of AAS, no publications have assessed which AAS has the greatest potential to cause this side effect or explain whether stanozolol is specifically related to orthopedic complications, as revealed in our study.

Demonstrating the possibility of these relationships is very significant from the perspective of endocrinology and reproductive medicine and constitutes a very significant contribution to these fields.

### 4.5. Potential Public Health Interventions to Prevent AAS Abuse

Despite the growing scale of AAS use in the world, recent reviews of illicit substance use prevention have found that preventive interventions are mainly school-based and focus on sports; moreover, there is a lack of good evidence on the effectiveness of interventions for banned substances outside competitive sports [69]. It was also shown that, although effective prevention of doping in recreational sports was considered significant in most countries in the European Union, research examining the effects of preventive education programs has been rare [70]. Two recent prevention programs deserve attention. Since 2008, a 100% pure heavy training (100% PHT) program has been running in Sweden, aimed at reducing the use of doping among people training recreationally at the gym. So far, it has covered over 500 gyms, gym employees, and owners; moreover, the police participate in the program, as well as authorities and municipal and provincial prevention coordinators. The program includes training gym staff and improving law enforcement. The effects of this program are currently being assessed [71]. The second program (Doping E-learning Tools (DELTS)) was implemented as part of the 2018–2019 Erasmus+ Sport project. Its goal was to improve preventive health education materials to counteract doping in recreational sports. This program evaluated e-learning tools aimed at healthcare providers and the fitness industry. The survey and interviews showed that all tools were clear and easy to use. The second main result of the project involved the implementation of a best-practice data bank [72]. There is a need to implement similar preventive programs, evaluate and improve them, and implement structured support strategies for the cessation of AAS use and legislative changes.

### 4.6. Limitations

Our work has some limitations, including narrowing the analysis to one database and a narrow range of keywords. These limitations result from the long observation period and the large amount of data already obtained as a result of this search method. Another is the fact that we analyzed published clinical cases; the number of these publications depends on many factors, including the publication activity of doctors treating the effects of AAS poisoning, whether a given clinical case may constitute an interesting report (and is accepted by the publisher), and whether illegal practices are not disclosed; therefore, the number of cases described does not necessarily reflect the actual number. Nevertheless, this work is the first attempt to assess the impact of illegal AAS use on both reproductive and social health, and the first attempt to demonstrate the relationship between the use of individual substances and specific adverse effects.

## 5. Conclusions

In our work, we demonstrated an increasing trend in the number of publications describing serious medical problems associated with the use of AAS, a trend of using several substances simultaneously, and preferential use of substances with a high potential to cause serious side effects. These behaviors concern people with an average age of about 30, i.e., people of reproductive age. The medical problems described, which dominate clinical case reports, include serious cardiological, psychiatric, endocrinological, hepatic, and oncological diseases, and although we cannot predict the outcomes with certainty, these problems may significantly affect the reproductive health of these people and pose a challenge for reproductive medicine. Moreover, they limit the social and professional functioning of these people and contribute to a growing healthcare burden.

## Figures and Tables

**Table 1:** The number of cases in which individual AAS were used during the period 1973–2023, divided into decades.

Substance	Period				
	1973–1982	1983–1992	1993–2002	2003–2012	2013–2022
1-androsterone	0	0	0	1	0
19-norandrosterone	0	1	0	3	0
3α-androstanediol	0	0	0	0	1
androstanolone	0	0	1	0	1
androstenediol	0	0	0	2	0
androstenedione	0	0	1	4	0
androsterone	1	0	0	2	0
boldenone	0	1	1	5	3
clenbuterol	0	0	4	2	2
danazol	2	1	3	1	0
dehydroepiandrosterone	0	0	5	4	0
deksametazon	0	0	0	0	0
drostanolone	0	0	1	2	3
epitestosterone	0	0	0	2	0
ethylestrenol	0	1	0	1	0
fluoxymesterone	3	2	1	3	2
mesterolone	1	2	2	5	3
methandienone	4	11	20	22	8
methasterone	0	0	0	2	0
methenolone	1	4	4	11	6
methylstenbolone	0	0	0	3	2
methyltestosterone	1	2	4	5	0
nandrolone	1	14	33	41	19
norethandrolone	0	1	1	0	0
not specified AAS	2	9	17	14	16
oxandrolone	0	6	1	4	4
oxymetholone	6	1	11	12	4
selective androgen receptor modulators	0	0	0	0	1
stanozolol	2	9	20	27	15
testosterone	5	27	46	79	38
trenbolone	0	1	1	11	10
Total	29	93	177	268	138

**Table 2:** The percentage of AAS used in the analyzed clinical cases during the period 1973–2023, divided into decades.

Substance	Period				
	1973–1982	1983–1992	1993–2002	2003–2012	2013–2022
1-androsterone	0.00	0.00	0.00	0.37	0.00
19-norandrosterone	0.00	1.08	0.00	1.12	0.00
3α-androstanediol	0.00	0.00	0.00	0.00	0.72
androstanolone	0.00	0.00	0.56	0.00	0.72
androstenediol	0.00	0.00	0.00	0.75	0.00
androstenedione	0.00	0.00	0.56	1.49	0.00
androsterone	3.45	0.00	0.00	0.75	0.00
boldenone	0.00	1.08	0.56	1.87	2.17
clenbuterol	0.00	0.00	2.26	0.75	1.45
danazol	6.90	1.08	1.69	0.37	0.00
dehydroepiandrosterone	0.00	0.00	2.82	1.49	0.00
deksametazon	0.00	0.00	0.00	0.00	0.00
drostanolone	0.00	0.00	0.56	0.75	2.17
epitestosterone	0.00	0.00	0.00	0.75	0.00
ethylestrenol	0.00	1.08	0.00	0.37	0.00
fluoxymesterone	10.34	2.15	0.56	1.12	1.45
mesterolone	3.45	2.15	1.13	1.87	2.17
methandienone	13.79	11.83	11.30	8.21	5.80
methasterone	0.00	0.00	0.00	0.75	0.00
methenolone	3.45	4.30	2.26	4.10	4.35
methylstenbolone	0.00	0.00	0.00	1.12	1.45
methyltestosterone	3.45	2.15	2.26	1.87	0.00
nandrolone	3.45	15.05	18.64	15.30	13.77
norethandrolone	0.00	1.08	0.56	0.00	0.00
not specified AAS	6.90	9.68	9.60	5.22	11.59
oxandrolone	0.00	6.45	0.56	1.49	2.90
oxymetholone	20.69	1.08	6.21	4.48	2.90
selective androgen receptor modulators	0.00	0.00	0.00	0.00	0.72
stanozolol	6.90	9.68	11.30	10.07	10.87
testosterone	17.24	29.03	25.99	29.48	27.54
trenbolone	0.00	1.08	0.56	4.10	7.25
Total	100.00	100.00	100.00	100.00	100.00

**Table 3:** The number of clinical cases with AAS types used individually in each decade of the period 1973–2022.

Substance	Period				
	1973–1982	1983–1992	1993–2002	2003–2012	2013–2022
1-androsterone	0	0	0	0	0
19-norandrosterone	0	0	0	0	0
3α-androstanediol	0	0	0	0	0
androstanolone	0	0	1	0	0
androstenediol	0	0	0	2	0
androstenedione	0	0	1	1	0
androsterone	1	0	0	0	0
boldenone	0	0	0	0	0
clenbuterol	0	0	1	0	0
danazol	2	1	3	1	0
dehydroepiandrosterone	0	0	5	2	0
deksametazon	0	0	0	0	0
drostanolone	0	0	0	0	0
epitestosterone	0	0	0	0	0
ethylestrenol	0	0	0	0	0
fluoxymesterone	2	0	0	0	0
mesterolone	1	0	0	0	0
methandienone	4	1	4	2	1
methasterone	0	0	0	1	0
methenolone	1	0	0	0	1
methylstenbolone	0	0	0	2	2
methyltestosterone	0	1	3	0	0
nandrolone	0	2	11	10	3
norethandrolone	0	0	0	0	0
not specified AAS	2	9	17	14	16
oxandrolone	0	0	0	0	1
oxymetholone	5	1	7	0	2
selective androgen receptor modulators	0	0	0	0	0
stanozolol	2	0	3	1	0
testosterone	3	18	26	43	14
trenbolone	0	0	0	0	0
Total	23	33	82	79	40

**Table 4:** The percentage of clinical cases with AAS types used individually in each decade of the period 1973–2022.

Substance	Period				
	1973–1982	1983–1992	1993–2002	2003–2012	2013–2022
1-androsterone	0.00	0.00	0.00	0.00	0.00
19-norandrosterone	0.00	0.00	0.00	0.00	0.00
3α-androstanediol	0.00	0.00	0.00	0.00	0.00
androstanolone	0.00	0.00	1.22	0.00	0.00
androstenediol	0.00	0.00	0.00	2.53	0.00
androstenedione	0.00	0.00	1.22	1.27	0.00
androsterone	4.35	0.00	0.00	0.00	0.00
boldenone	0.00	0.00	0.00	0.00	0.00
clenbuterol	0.00	0.00	1.22	0.00	0.00
danazol	8.70	3.03	3.66	1.27	0.00
dehydroepiandrosterone	0.00	0.00	6.10	2.53	0.00
deksametazon	0.00	0.00	0.00	0.00	0.00
drostanolone	0.00	0.00	0.00	0.00	0.00
epitestosterone	0.00	0.00	0.00	0.00	0.00
ethylestrenol	0.00	0.00	0.00	0.00	0.00
fluoxymesterone	8.70	0.00	0.00	0.00	0.00
mesterolone	4.35	0.00	0.00	0.00	0.00
methandienone	17.39	3.03	4.88	2.53	2.50
methasterone	0.00	0.00	0.00	1.27	0.00
methenolone	4.35	0.00	0.00	0.00	2.50
methylstenbolone	0.00	0.00	0.00	2.53	5.00
methyltestosterone	0.00	3.03	3.66	0.00	0.00
nandrolone	0.00	6.06	13.41	12.66	7.50
norethandrolone	0.00	0.00	0.00	0.00	0.00
not specified AAS	8.70	27.27	20.73	17.72	40.00
oxandrolone	0.00	0.00	0.00	0.00	2.50
oxymetholone	21.74	3.03	8.54	0.00	5.00
selective androgen receptor modulators	0.00	0.00	0.00	0.00	0.00
stanozolol	8.70	0.00	3.66	1.27	0.00
testosterone	13.04	54.55	31.71	54.43	35.00
trenbolone	0.00	0.00	0.00	0.00	0.00
Total	100.00	100.00	100.00	100.00	100.00

**Table 5:** The number of clinical cases with AAS types used in combination with several substances in each decade of the period 1973–2022.

Substance	Period				
	1973–1982	1983–1992	1993–2002	2003–2012	2013–2022
1-androsterone	0	0	0	1	0
19-norandrosterone	0	1	0	3	0
3α-androstanediol	0	0	0	0	1
androstanolone	0	0	0	0	1
androstenediol	0	0	0	0	0
androstenedione	0	0	0	3	0
androsterone	0	0	0	2	0
boldenone	0	1	1	5	3
clenbuterol	0	0	3	2	2
danazol	0	0	0	0	0
dehydroepiandrosterone	0	0	0	2	0
deksametazon	0	0	0	0	0
drostanolone	0	0	1	2	3
epitestosterone	0	0	0	2	0
ethylestrenol	0	1	0	1	0
fluoxymesterone	1	2	1	3	2
mesterolone	0	2	2	5	3
methandienone	0	10	16	20	7
methasterone	0	0	0	1	0
methenolone	0	4	4	11	5
methylstenbolone	0	0	0	1	0
methyltestosterone	1	1	1	5	0
nandrolone	1	12	22	31	16
norethandrolone	0	1	1	0	0
not specified AAS	0	0	0	0	0
oxandrolone	0	6	1	4	3
oxymetholone	1	0	4	12	2
selective androgen receptor modulators	0	0	0	0	1
stanozolol	0	9	17	26	15
testosterone	2	9	20	36	24
trenbolone	0	1	1	11	10
Total	6	60	95	189	98

**Table 6:** The percentage of clinical cases with AAS types used in combination with several substances in each decade of the period 1973–2022.

Substance	Period				
	1973–1982	1983–1992	1993–2002	2003–2012	2013–2022
1-androsterone	0.00	0.00	0.00	0.53	0.00
19-norandrosterone	0.00	1.67	0.00	1.59	0.00
3α-androstanediol	0.00	0.00	0.00	0.00	1.02
androstanolone	0.00	0.00	0.00	0.00	1.02
androstenediol	0.00	0.00	0.00	0.00	0.00
androstenedione	0.00	0.00	0.00	1.59	0.00
androsterone	0.00	0.00	0.00	1.06	0.00
boldenone	0.00	1.67	1.05	2.65	3.06
clenbuterol	0.00	0.00	3.16	1.06	2.04
danazol	0.00	0.00	0.00	0.00	0.00
dehydroepiandrosterone	0.00	0.00	0.00	1.06	0.00
deksametazon	0.00	0.00	0.00	0.00	0.00
drostanolone	0.00	0.00	1.05	1.06	3.06
epitestosterone	0.00	0.00	0.00	1.06	0.00
ethylestrenol	0.00	1.67	0.00	0.53	0.00
fluoxymesterone	16.67	3.33	1.05	1.59	2.04
mesterolone	0.00	3.33	2.11	2.65	3.06
methandienone	0.00	16.67	16.84	10.58	7.14
methasterone	0.00	0.00	0.00	0.53	0.00
methenolone	0.00	6.67	4.21	5.82	5.10
methylstenbolone	0.00	0.00	0.00	0.53	0.00
methyltestosterone	16.67	1.67	1.05	2.65	0.00
nandrolone	16.67	20.00	23.16	16.40	16.33
norethandrolone	0.00	1.67	1.05	0.00	0.00
not specified AAS	0.00	0.00	0.00	0.00	0.00
oxandrolone	0.00	10.00	1.05	2.12	3.06
oxymetholone	16.67	0.00	4.21	6.35	2.04
selective androgen receptor modulators	0.00	0.00	0.00	0.00	1.02
stanozolol	0.00	15.00	17.89	13.76	15.31
testosterone	33.33	15.00	21.05	19.05	24.49
trenbolone	0.00	1.67	1.05	5.82	10.20
Total	100.00	100.00	100.00	100.00	100.00

**Table 7:** The types of medical problems among all cases.

Type of Medical Problem	N	%
cardiological	69	17.4
psychiatric	56	14.1
endocrinological	45	11.3
hepatological	44	11.1
oncological	35	8.8
dermatological	33	8.3
andrological	32	8.1
neurological	23	5.8
infectious	19	4.8
hemorrhagic	18	4.5
orthopedical	15	3.8
thromboembolic	15	3.8
hematological	13	3.3
pulmonological	7	1.8
rhabdomyolysis	7	1.8
hypertension	6	1.5
metabolic	5	1.3
nephrological	4	1.0
otolaryngological	4	1.0
urological	4	1.0
gastroenterological	3	0.8
gynecological	3	0.8
ophtalmologic	2	0.5
diabetological	1	0.3
myocardial infarction	1	0.3
pharmacological	1	0.3
sexological	1	0.3
surgical	1	0.3

**Table 8:** The number of different types of side effects related to the use of AAS in each decade of the period 1973–2022.

Type of Medical Problem	Period					Total
	1973–1982	1983–1992	1993–2002	2003–2012	2013–2022	
andrological	0	6	12	14	0	32
cardiological	0	4	20	20	25	69
dermatological	1	8	12	10	2	33
diabetological	0	0	0	0	1	1
endocrinological	3	4	10	25	3	45
gastroenterological	0	0	0	0	3	3
gynecological	1	0	0	2	0	3
hematological	0	0	6	6	1	13
hemorrhagic	3	2	4	6	3	18
hepatological	9	4	8	12	11	44
hypertension	0	1	1	4	0	6
infectious	0	1	6	10	2	19
metabolic	0	1	1	1	2	5
myocardial infarction	0	0	0	1	0	1
nephrological	0	0	2	1	1	4
neurological	1	3	4	9	6	23
oncological	5	3	13	8	6	35
ophtalmologic	0	0	0	2	0	2
orthopedical	1	2	9	3	0	15
otolaryngological	0	0	1	2	1	4
pharmacological	0	1	0	0	0	1
psychiatric	0	8	20	22	6	56
pulmonological	0	4	2	1	0	7
rhabdomyolysis	0	1	2	4	0	7
sexological	0	0	0	1	0	1
surgical	0	0	0	0	1	1
thromboembolic	4	2	1	4	4	15
urological	1	1	2	0	0	4
Total	29	56	136	168	78	467

**Table 9:** The percentage of types of side effects related to the use of AAS in each decade of the period 1973–2022.

Type of Medical Problem	Period				
	1973–1982	1983–1992	1993–2002	2003–2012	2013–2022
andrological	0.0	10.7	8.8	8.3	0.0
cardiological	0.0	7.1	14.7	11.9	32.1
dermatological	3.4	14.3	8.8	6.0	2.6
diabetological	0.0	0.0	0.0	0.0	1.3
endocrinological	10.3	7.1	7.4	14.9	3.8
gastroenterological	0.0	0.0	0.0	0.0	3.8
gynecological	3.4	0.0	0.0	1.2	0.0
hematological	0.0	0.0	4.4	3.6	1.3
hemorrhagic	10.3	3.6	2.9	3.6	3.8
hepatological	31.0	7.1	5.9	7.1	14.1
hypertension	0.0	1.8	0.7	2.4	0.0
infectious	0.0	1.8	4.4	6.0	2.6
metabolic	0.0	1.8	0.7	0.6	2.6
myocardial infarction	0.0	0.0	0.0	0.6	0.0
nephrological	0.0	0.0	1.5	0.6	1.3
neurological	3.4	5.4	2.9	5.4	7.7
oncological	17.2	5.4	9.6	4.8	7.7
ophtalmologic	0.0	0.0	0.0	1.2	0.0
orthopedical	3.4	3.6	6.6	1.8	0.0
otolaryngological	0.0	0.0	0.7	1.2	1.3
pharmacological	0.0	1.8	0.0	0.0	0.0
psychiatric	0.0	14.3	14.7	13.1	7.7
pulmonological	0.0	7.1	1.5	0.6	0.0
rhabdomyolysis	0.0	1.8	1.5	2.4	0.0
sexological	0.0	0.0	0.0	0.6	0.0
surgical	0.0	0.0	0.0	0.0	1.3
thromboembolic	13.8	3.6	0.7	2.4	5.1
urological	3.4	1.8	1.5	0.0	0.0
Total	100.0	100.0	100.0	100.0	100.0

**Table 10:** The share of individual diseases among all cases.

Disease Entity	N	%
Myocardial infarction	25	11.0
Virilisation	19	8.4
Cholestasis	18	7.9
Hypertrophic cardiomyopathy	17	7.5
Liver cancer	15	6.6
Male infertility	15	6.6
Dilated cardiomyopathy	9	4.0
Liver toxicity	8	3.5
Local infection	8	3.5
Prostate cancer	8	3.5
Arrhythmia	7	3.1
Priapism	7	3.1
Liver enlargement	5	2.2
Peliosis of the liver	4	1.8
Gynecomastia	4	1.8
Liver tumor	4	1.8
Testicular cancer	4	1.8
Tuberculosis	3	1.3
Impotence	3	1.3
HIV, Hepatitis B	3	1.3
Gynecomastia, testicular athrophy	3	1.3
Testicular athrophy	3	1.3
Other	2	0.9
HIV	2	0.9
Pituitary tumor	2	0.9
Cardiogenic shock	2	0.9
Premature puberty	2	0.9
HIV, Erectile dysfunction	2	0.9
Pituitary enlrgement	1	0.4
Lymphoma	1	0.4
Liver rapture	1	0.4
Kidney cancer	1	0.4
Myocarditis	1	0.4
Colon cancer	1	0.4
Wilms tumor	1	0.4
Hypertrophic cardiomyopathy, myocardial infarction	1	0.4
Pseudohermaphroditism	1	0.4
Dilated cardiomyopathy, Liver toxicity	1	0.4
Pituitary dysfunction	1	0.4
Covid-19	1	0.4
Spontaneous coronary artery dissection	1	0.4
Hypertrophic cardiomyopathy, Liver enlargement	1	0.4
Hypoglycemia	1	0.4

**Table 11:** Statistically significant relationships between the given types of side effects and the type of AAS, when used individually.

Type of Side Effect	Type of AAS	*p*	P adj.	Occurrence (%)	
				In Non-Users	In Users
andrological	androstenedione	0.0058	0.2233	7.1	100.0
cardiological	nandrolone	0.0279	0.6406	10.8	26.9
dermatological	testosterone	0.0281	0.6406	5.9	14.4
endocrinological	not specified AAS	0.0017	0.0873	17.1	1.7
	testosterone	0.0000	0.0000	5.9	25.0
hematological	danazol	0.0312	0.6406	3.6	28.6
hemorrhagic	methandienone	0.0078	0.2669	2.9	25.0
hepatological	methylstenbolone	0.0001	0.0154	8.7	100.0
	oxymetholone	0.0016	0.0873	8.3	40.0
	testosterone	0.0013	0.0873	15.0	2.9
nephrological	methylstenbolone	0.0309	0.6406	0.4	25.0
	stanozolol	0.0462	0.7084	0.4	16.7
neurological	dehydroepiandrosterone	0.0496	0.7084	4.8	28.6
oncological	methyltestosterone	0.0035	0.1540	9.1	75.0
orthopedical	stanozolol	0.0189	0.5292	3.2	33.3
psychiatric	dehydroepiandrosterone	0.0435	0.7084	11.6	42.9
	methandienone	0.0092	0.2834	11.0	41.7
thromboembolic	fluoxymesterone	0.0006	0.0616	2.0	100.0
	testosterone	0.0438	0.7084	4.6	0.0

**Table 12:** Statistically significant relationships between the given types of side effects and the use of several AAS types at the same time.

Type of Side Effect	*p*	P adj.
andrological	0.8010	1.0000
cardiological	0.0021	0.0588
dermatological	0.4452	0.8904
diabetological	0.3526	0.8227
endocrinological	0.0256	0.3332
gastroenterological	0.7298	1.0000
genecological	1.0000	1.0000
hematological	0.2211	0.7739
hemorrhagic	0.5566	0.9741
hepatological	0.6707	0.9884
hypertension	0.5935	0.9775
infectious	0.6545	0.9884
metabolic	0.0357	0.3332
myocardial infarction	1.0000	1.0000
nephrological	0.2837	0.7944
neurological	0.1922	0.7688
oncological	0.5166	0.9643
ophtalmologic	1.0000	1.0000
orthopedical	0.3957	0.8523
otolaryngological	1.0000	1.0000
pharmacological	1.0000	1.0000
psychiatric	0.0888	0.4973
pulmonological	0.2593	0.7944
rhabdomyolysis	0.0733	0.4973
sexological	1.0000	1.0000
surgical	0.3526	0.8227
thromboembolic	0.1068	0.4984
urological	1.0000	1.0000

## Data Availability

Upon request from the corresponding author.

## Supplementary Materials

The following supporting information was downloaded at: https://www.mdpi.com/article/10.3390/jcm13195892/s1, Table S1: The relationships between the given types of side effects and the type of AAS when used individually.
