# Supplementary material for: Abuse of Anabolic-Androgenic Steroids as a Social Phenomenon and Medical Problem—Its Potential Negative Impact on Reproductive Health Based on 50 Years of Case Report Analysis
Source: J Clin Med. 2024 Oct 2;13(19):5892. doi: 10.3390/jcm13195892 (PMC11478083; doi:10.3390/jcm13195892)
Supplement: Supplementary file 1 [file jcm-13-05892-s001.zip › jcm-3210592-supplementary.pdf]

Table S1. The relationship between the occurrence of specific side effects and the type of AAS used individually. The occurrence (%) is calculated relative to the number of patients experiencing each type of side effect. P adj. indicates the p-value calculated using the Benjamini-Hochberg correction for controlling Type I error.

Type of side effect	Type of AAS	P	P adj.	Occurrence (%)	
				In non-users	In users
andrological	androstenediol	1.000	1.000	7.8	0.0
	androstenedione	0.006	0.223	7.1	100.0
	danazol	0.437	1.000	7.6	14.3
	dehydroepiandrosterone	1.000	1.000	8.0	0.0
	fluoxymesterone	1.000	1.000	7.8	0.0
	methandienone	0.607	1.000	8.2	0.0
	methenolone	1.000	1.000	7.8	0.0
	methylstenbolone	1.000	1.000	7.9	0.0
	methyltestosterone	1.000	1.000	7.9	0.0
	nandrolone	1.000	1.000	7.8	7.7
	not specified AAS	0.783	1.000	7.5	8.6
	oxymetholone	0.615	1.000	8.3	0.0
	stanozolol	1.000	1.000	8.0	0.0
	testosterone	0.477	1.000	6.5	9.6
cardiological	androstenediol	1.000	1.000	12.5	0.0
	androstenedione	1.000	1.000	12.5	0.0
	danazol	0.602	1.000	12.8	0.0
	dehydroepiandrosterone	1.000	1.000	12.4	14.3
	fluoxymesterone	1.000	1.000	12.5	0.0
	methandienone	1.000	1.000	12.7	8.3
	methenolone	0.234	1.000	12.2	50.0
	methylstenbolone	1.000	1.000	12.6	0.0
	methyltestosterone	1.000	1.000	12.6	0.0
	nandrolone	0.028	0.641	10.8	26.9
	not specified AAS	0.257	1.000	11.1	17.2
	oxymetholone	0.228	1.000	13.2	0.0
	stanozolol	1.000	1.000	12.7	0.0
	testosterone	0.565	1.000	13.7	10.6
dermatological	androstenediol	1.000	1.000	9.4	0.0
	androstenedione	1.000	1.000	9.4	0.0
	danazol	1.000	1.000	9.6	0.0
	dehydroepiandrosterone	1.000	1.000	9.6	0.0
	fluoxymesterone	1.000	1.000	9.4	0.0
	methandienone	0.611	1.000	9.8	0.0
	methenolone	1.000	1.000	9.4	0.0
	methylstenbolone	0.326	1.000	9.1	25.0
	methyltestosterone	1.000	1.000	9.5	0.0

	nandrolone	0.281	1.000	8.7	15.4
	not specified AAS	0.121	0.995	11.1	3.4
	oxymetholone	1.000	1.000	9.5	6.7
	stanozolol	1.000	1.000	9.6	0.0
	testosterone	0.028	0.641	5.9	14.4
endocrinological	androstenediol	0.254	1.000	13.3	50.0
	androstenedione	1.000	1.000	13.7	0.0
	danazol	0.055	0.708	12.8	42.9
	dehydroepiandrosterone	0.598	1.000	14.0	0.0
	fluoxymesterone	1.000	1.000	13.7	0.0
	methandienone	0.380	1.000	14.3	0.0
	methenolone	1.000	1.000	13.7	0.0
	methylstenbolone	1.000	1.000	13.8	0.0
	methyltestosterone	1.000	1.000	13.8	0.0
	nandrolone	0.764	1.000	13.4	15.4
	not specified AAS	0.002	0.087	17.1	1.7
	oxymetholone	0.235	1.000	14.5	0.0
	stanozolol	1.000	1.000	13.9	0.0
	testosterone	0.000	0.000	5.9	25.0
gastroenterological	androstenediol	1.000	1.000	0.8	0.0
	androstenedione	1.000	1.000	0.8	0.0
	danazol	1.000	1.000	0.8	0.0
	dehydroepiandrosterone	1.000	1.000	0.8	0.0
	fluoxymesterone	1.000	1.000	0.8	0.0
	methandienone	1.000	1.000	0.8	0.0
	methenolone	1.000	1.000	0.8	0.0
	methylstenbolone	1.000	1.000	0.8	0.0
	methyltestosterone	1.000	1.000	0.8	0.0
	nandrolone	1.000	1.000	0.9	0.0
	not specified AAS	0.401	1.000	0.5	1.7
	oxymetholone	0.114	0.981	0.4	6.7
	stanozolol	1.000	1.000	0.8	0.0
	testosterone	0.516	1.000	1.3	0.0
gynecological	androstenediol	1.000	1.000	1.2	0.0
	androstenedione	1.000	1.000	1.2	0.0
	danazol	1.000	1.000	1.2	0.0
	dehydroepiandrosterone	1.000	1.000	1.2	0.0
	fluoxymesterone	1.000	1.000	1.2	0.0
	methandienone	1.000	1.000	1.2	0.0
	methenolone	1.000	1.000	1.2	0.0
	methylstenbolone	1.000	1.000	1.2	0.0
	methyltestosterone	1.000	1.000	1.2	0.0
	nandrolone	1.000	1.000	1.3	0.0
	not specified AAS	1.000	1.000	1.5	0.0
	oxymetholone	1.000	1.000	1.2	0.0
	stanozolol	1.000	1.000	1.2	0.0

	testosterone	0.065	0.771	0.0	2.9
hematological	androstenediol	1.000	1.000	4.3	0.0
	androstenedione	1.000	1.000	4.3	0.0
	danazol	0.031	0.641	3.6	28.6
	dehydroepiandrosterone	1.000	1.000	4.4	0.0
	fluoxymesterone	1.000	1.000	4.3	0.0
	methandienone	0.415	1.000	4.1	8.3
	methenolone	1.000	1.000	4.3	0.0
	methylstenbolone	1.000	1.000	4.3	0.0
	methyltestosterone	1.000	1.000	4.3	0.0
	nandrolone	1.000	1.000	4.3	3.8
	not specified AAS	0.131	0.999	5.5	0.0
	oxymetholone	1.000	1.000	4.5	0.0
	stanozolol	1.000	1.000	4.4	0.0
	testosterone	0.360	1.000	3.3	5.8
hemorrhagic	androstenediol	1.000	1.000	3.9	0.0
	androstenedione	1.000	1.000	3.9	0.0
	danazol	0.245	1.000	3.6	14.3
	dehydroepiandrosterone	1.000	1.000	4.0	0.0
	fluoxymesterone	1.000	1.000	3.9	0.0
	methandienone	0.008	0.267	2.9	25.0
	methenolone	1.000	1.000	3.9	0.0
	methylstenbolone	1.000	1.000	4.0	0.0
	methyltestosterone	1.000	1.000	4.0	0.0
	nandrolone	0.605	1.000	4.3	0.0
	not specified AAS	0.699	1.000	3.5	5.2
	oxymetholone	0.109	0.981	3.3	13.3
	stanozolol	1.000	1.000	4.0	0.0
	testosterone	0.053	0.708	5.9	1.0
hepatological	androstenediol	1.000	1.000	10.2	0.0
	androstenedione	1.000	1.000	10.2	0.0
	danazol	1.000	1.000	10.4	0.0
	dehydroepiandrosterone	0.151	1.000	9.6	28.6
	fluoxymesterone	1.000	1.000	10.2	0.0
	methandienone	0.347	1.000	9.8	16.7
	methenolone	1.000	1.000	10.2	0.0
	methylstenbolone	0.000	0.015	8.7	100.0
	methyltestosterone	1.000	1.000	10.3	0.0
	nandrolone	0.087	0.836	11.3	0.0
	not specified AAS	1.000	1.000	10.1	10.3
	oxymetholone	0.002	0.087	8.3	40.0
	stanozolol	0.115	0.981	9.6	33.3
	testosterone	0.001	0.087	15.0	2.9
hypertension	androstenediol	1.000	1.000	1.2	0.0
	androstenedione	1.000	1.000	1.2	0.0
	danazol	1.000	1.000	1.2	0.0

	dehydroepiandrosterone	1.000	1.000	1.2	0.0
	fluoxymesterone	1.000	1.000	1.2	0.0
	methandienone	1.000	1.000	1.2	0.0
	methenolone	1.000	1.000	1.2	0.0
	methylstenbolone	1.000	1.000	1.2	0.0
	methyltestosterone	1.000	1.000	1.2	0.0
	nandrolone	1.000	1.000	1.3	0.0
	not specified AAS	1.000	1.000	1.5	0.0
	oxymetholone	1.000	1.000	1.2	0.0
	stanozolol	1.000	1.000	1.2	0.0
	testosterone	0.065	0.771	0.0	2.9
infectious	androstenediol	1.000	1.000	4.3	0.0
	androstenedione	1.000	1.000	4.3	0.0
	danazol	1.000	1.000	4.4	0.0
	dehydroepiandrosterone	1.000	1.000	4.4	0.0
	fluoxymesterone	1.000	1.000	4.3	0.0
	methandienone	1.000	1.000	4.5	0.0
	methenolone	1.000	1.000	4.3	0.0
	methylstenbolone	1.000	1.000	4.3	0.0
	methyltestosterone	1.000	1.000	4.3	0.0
	nandrolone	0.308	1.000	3.9	7.7
	not specified AAS	0.075	0.813	3.0	8.6
	oxymetholone	1.000	1.000	4.5	0.0
	stanozolol	0.233	1.000	4.0	16.7
	testosterone	0.533	1.000	5.2	2.9
nephrological	androstenediol	1.000	1.000	0.8	0.0
	androstenedione	1.000	1.000	0.8	0.0
	danazol	1.000	1.000	0.8	0.0
	dehydroepiandrosterone	1.000	1.000	0.8	0.0
	fluoxymesterone	1.000	1.000	0.8	0.0
	methandienone	1.000	1.000	0.8	0.0
	methenolone	1.000	1.000	0.8	0.0
	methylstenbolone	0.031	0.641	0.4	25.0
	methyltestosterone	1.000	1.000	0.8	0.0
	nandrolone	1.000	1.000	0.9	0.0
	not specified AAS	1.000	1.000	1.0	0.0
	oxymetholone	1.000	1.000	0.8	0.0
	stanozolol	0.046	0.708	0.4	16.7
	testosterone	0.516	1.000	1.3	0.0
neurological	androstenediol	1.000	1.000	5.5	0.0
	androstenedione	1.000	1.000	5.5	0.0
	danazol	1.000	1.000	5.6	0.0
	dehydroepiandrosterone	0.050	0.708	4.8	28.6
	fluoxymesterone	1.000	1.000	5.5	0.0
	methandienone	0.133	0.999	4.9	16.7
	methenolone	1.000	1.000	5.5	0.0

	methylstenbolone	1.000	1.000	5.5	0.0
	methyltestosterone	1.000	1.000	5.5	0.0
	nandrolone	0.640	1.000	5.2	7.7
	not specified AAS	0.742	1.000	6.0	3.4
	oxymetholone	0.579	1.000	5.4	6.7
	stanozolol	0.288	1.000	5.2	16.7
	testosterone	0.168	1.000	7.2	2.9
oncological	androstenediol	1.000	1.000	10.2	0.0
	androstenedione	1.000	1.000	10.2	0.0
	danazol	0.530	1.000	10.0	14.3
	dehydroepiandrosterone	1.000	1.000	10.4	0.0
	fluoxymesterone	1.000	1.000	10.2	0.0
	methandienone	0.109	0.981	9.4	25.0
	methenolone	1.000	1.000	10.2	0.0
	methylstenbolone	1.000	1.000	10.3	0.0
	methyltestosterone	0.004	0.154	9.1	75.0
	nandrolone	0.087	0.836	11.3	0.0
	not specified AAS	0.462	1.000	11.1	6.9
	oxymetholone	0.052	0.708	9.1	26.7
	stanozolol	1.000	1.000	10.4	0.0
	testosterone	0.836	1.000	9.8	10.6
ophtalmologic	androstenediol	1.000	1.000	0.8	0.0
	androstenedione	1.000	1.000	0.8	0.0
	danazol	1.000	1.000	0.8	0.0
	dehydroepiandrosterone	1.000	1.000	0.8	0.0
	fluoxymesterone	1.000	1.000	0.8	0.0
	methandienone	1.000	1.000	0.8	0.0
	methenolone	1.000	1.000	0.8	0.0
	methylstenbolone	1.000	1.000	0.8	0.0
	methyltestosterone	1.000	1.000	0.8	0.0
	nandrolone	0.193	1.000	0.4	3.8
	not specified AAS	1.000	1.000	1.0	0.0
	oxymetholone	1.000	1.000	0.8	0.0
	stanozolol	1.000	1.000	0.8	0.0
	testosterone	1.000	1.000	0.7	1.0
orthopedical	androstenediol	0.077	0.813	3.5	50.0
	androstenedione	1.000	1.000	3.9	0.0
	danazol	1.000	1.000	4.0	0.0
	dehydroepiandrosterone	1.000	1.000	4.0	0.0
	fluoxymesterone	1.000	1.000	3.9	0.0
	methandienone	1.000	1.000	4.1	0.0
	methenolone	1.000	1.000	3.9	0.0
	methylstenbolone	1.000	1.000	4.0	0.0
	methyltestosterone	1.000	1.000	4.0	0.0
	nandrolone	0.605	1.000	4.3	0.0
	not specified AAS	0.240	1.000	3.0	6.9

	oxymetholone	0.458	1.000	3.7	6.7
	stanozolol	0.019	0.529	3.2	33.3
	testosterone	0.210	1.000	5.2	1.9
otolaryngological	androstenediol	1.000	1.000	1.2	0.0
	androstenedione	1.000	1.000	1.2	0.0
	danazol	1.000	1.000	1.2	0.0
	dehydroepiandrosterone	1.000	1.000	1.2	0.0
	fluoxymesterone	1.000	1.000	1.2	0.0
	methandienone	1.000	1.000	1.2	0.0
	methenolone	1.000	1.000	1.2	0.0
	methylstenbolone	1.000	1.000	1.2	0.0
	methyltestosterone	1.000	1.000	1.2	0.0
	nandrolone	0.275	1.000	0.9	3.8
	not specified AAS	0.129	0.999	0.5	3.4
	oxymetholone	1.000	1.000	1.2	0.0
	stanozolol	1.000	1.000	1.2	0.0
	testosterone	0.274	1.000	2.0	0.0
psychiatric	androstenediol	1.000	1.000	12.5	0.0
	androstenedione	1.000	1.000	12.5	0.0
	danazol	0.602	1.000	12.8	0.0
	dehydroepiandrosterone	0.044	0.708	11.6	42.9
	fluoxymesterone	1.000	1.000	12.5	0.0
	methandienone	0.009	0.283	11.0	41.7
	methenolone	1.000	1.000	12.5	0.0
	methylstenbolone	1.000	1.000	12.6	0.0
	methyltestosterone	0.415	1.000	12.3	25.0
	nandrolone	0.544	1.000	12.1	15.4
	not specified AAS	1.000	1.000	12.6	12.1
	oxymetholone	0.228	1.000	13.2	0.0
	stanozolol	1.000	1.000	12.7	0.0
	testosterone	0.848	1.000	13.1	11.5
pulmonological	androstenediol	1.000	1.000	2.7	0.0
	androstenedione	1.000	1.000	2.7	0.0
	danazol	1.000	1.000	2.8	0.0
	dehydroepiandrosterone	1.000	1.000	2.8	0.0
	fluoxymesterone	1.000	1.000	2.7	0.0
	methandienone	1.000	1.000	2.9	0.0
	methenolone	1.000	1.000	2.7	0.0
	methylstenbolone	1.000	1.000	2.8	0.0
	methyltestosterone	1.000	1.000	2.8	0.0
	nandrolone	1.000	1.000	3.0	0.0
	not specified AAS	0.657	1.000	2.5	3.4
	oxymetholone	1.000	1.000	2.9	0.0
	stanozolol	1.000	1.000	2.8	0.0
	testosterone	0.123	0.995	1.3	4.8
rhabdomyolysis	androstenediol	1.000	1.000	0.8	0.0

	androstenedione	1.000	1.000	0.8	0.0
	danazol	1.000	1.000	0.8	0.0
	dehydroepiandrosterone	1.000	1.000	0.8	0.0
	fluoxymesterone	1.000	1.000	0.8	0.0
	methandienone	1.000	1.000	0.8	0.0
	methenolone	1.000	1.000	0.8	0.0
	methylstenbolone	1.000	1.000	0.8	0.0
	methyltestosterone	1.000	1.000	0.8	0.0
	nandrolone	1.000	1.000	0.9	0.0
	not specified AAS	0.050	0.708	0.0	3.4
	oxymetholone	1.000	1.000	0.8	0.0
	stanozolol	1.000	1.000	0.8	0.0
	testosterone	0.516	1.000	1.3	0.0
thromboembolic	androstenediol	1.000	1.000	2.7	0.0
	androstenedione	1.000	1.000	2.7	0.0
	danazol	1.000	1.000	2.8	0.0
	dehydroepiandrosterone	1.000	1.000	2.8	0.0
	fluoxymesterone	0.001	0.062	2.0	100.0
	methandienone	1.000	1.000	2.9	0.0
	methenolone	0.054	0.708	2.4	50.0
	methylstenbolone	1.000	1.000	2.8	0.0
	methyltestosterone	1.000	1.000	2.8	0.0
	nandrolone	0.530	1.000	2.6	3.8
	not specified AAS	0.657	1.000	2.5	3.4
	oxymetholone	1.000	1.000	2.9	0.0
	stanozolol	1.000	1.000	2.8	0.0
	testosterone	0.044	0.708	4.6	0.0
urological	androstenediol	1.000	1.000	1.2	0.0
	androstenedione	1.000	1.000	1.2	0.0
	danazol	1.000	1.000	1.2	0.0
	dehydroepiandrosterone	0.080	0.819	0.8	14.3
	fluoxymesterone	1.000	1.000	1.2	0.0
	methandienone	1.000	1.000	1.2	0.0
	methenolone	1.000	1.000	1.2	0.0
	methylstenbolone	1.000	1.000	1.2	0.0
	methyltestosterone	1.000	1.000	1.2	0.0
	nandrolone	1.000	1.000	1.3	0.0
	not specified AAS	1.000	1.000	1.5	0.0
	oxymetholone	1.000	1.000	1.2	0.0
	stanozolol	0.069	0.784	0.8	16.7
	testosterone	1.000	1.000	1.3	1.0
